# The role of blended learning in improving medical students’ academic performance: evidence from Pakistan

**DOI:** 10.3389/fmed.2024.1425659

**Published:** 2024-12-17

**Authors:** Muhammad Azeem Ashraf, Samson Maekele Tsegay, Nida Gull, Muneeba Saeed, Hussain Dawood

**Affiliations:** ^1^Institute of Educational Sciences, Hunan University, Changsha, China; ^2^School of Education, Anglia Ruskin University, Cambridge, United Kingdom; ^3^Business School, Hunan University, Changsha, China; ^4^School of Management Sciences, Quaid-i-Azam University, Islamabad, Pakistan; ^5^School of Computing, Skyline University College, Sharjah, United Arab Emirates

**Keywords:** blended learning, self-regulated learning, academic performance, technological competence, teachers’ credibility, perceived institutional support, medical education

## Abstract

**Objective:**

The study examines the role of blended learning in improving medical students’ academic performance through self-regulatory learning and technological competence and identifies the moderating role of perceived institutional support in the relationships between self-regulatory learning, perceived teacher credibility, technological competencies, and academic performance.

**Methods:**

The study was based on behavioral learning theory as a theoretical framework, and an adapted questionnaire was used to collect the data. In total, 275 medical students participated in the study, and the data was analyzed using structural equation modeling techniques with SmartPLS.

**Results:**

The results indicate that self-regulatory learning significantly affects student academic performance and mediates the role of teachers’ credibility and technological competencies. Furthermore, perceived institutional support is a significant moderator in the relationship between self-regulated learning, technological competencies, and teacher credibility.

**Conclusion:**

The study highlights the importance of self-regulated learning in students’ academic achievement. Moreover, it suggests that educational institutions should advance teachers’ competence and encourage collaborative learning to enhance students’ learning, motivation, and academic performance.

## Introduction

The impact of blended learning on medical students’ performance is complex, as it can cause positive or negative consequences depending on factors such as teachers’ technological and teaching skills (1–3). The complex nature of blended learning is more visible in medical education, a profession that might need more face-to-face contact and practical work experiences. Yet, with the advancement of information and communication technologies (ICT), the rapid institutional transition to blended learning has created new possibilities and challenges for students’ academic development (4, 5). Blended learning allowed students to balance academic and extra-curricular responsibilities (6, 7). Dziuban and Picciano (8) emphasized that participating in blended learning lectures and making use of digital resources help medical students develop the technological skills required to manage and cope with today’s data-driven society. In addition, blended learning promotes self-regulatory learning behavior of students accountable for their development and attendance (2).

Blended learning strategies have been extensively studied in education, resulting in numerous and some contradicting research findings (9, 10). Nevertheless, less attention has been given to research on self-regulatory learning under blended learning (3, 11). Gómez et al. (12) describe self-regulatory learning as a dynamic process in which learners establish learning goals and consciously monitor, regulate, and control their cognition, intentions, and behavior. Research also shows that teachers’ effectiveness is the most critical factor in shaping learners’ achievements (13) and influencing their academic performance (14). Several studies have explored effective teacher characteristics and concluded that teacher quality is the strongest predictor of academic performance (15).

In addition, research further suggests that instructors should give students enough direction and support to grasp and successfully engage in blended learning classes (16). Instructors must provide students with adequate direction and support to effectively engage in blended learning environments. This requirement highlights the importance of instructors and students possessing strong technological skills to participate in blended classrooms successfully (7). Based on these findings, this study investigates the effect of self-regulated learning on students’ academic performance in blended learning environments, precisely in medical education. Additionally, this research aims to identify the features and approaches that facilitate effective blended instruction and promote positive student outcomes.

Existing research underscores the significance of teachers’ credibility—defined as students’ belief in their ability to learn from a given teacher—and technological competence in motivating students and optimizing the use of blended learning (17–19). In this context, perceived institutional support, such as teachers’ credibility and technical skills, strongly predicts students’ satisfaction and commitment to self-regulatory learning (4, 20). Medical education demands highly skilled educators who can effectively integrate theoretical knowledge with practical application, shaping students’ expectations, motivation, and satisfaction (7).

While blended learning has been shown to enhance educational outcomes (18, 19), it has also raised concerns about the academic performance of medical students. However, adopting a learner-centered approach, supported by empirical evidence, has been found to positively impact educational achievements in blended learning environments (21). Masnadi (22) further emphasizes that, like other disciplines, medical science education requires competent and credible teachers to implement successful blended learning strategies. These observations suggest that multiple factors influence the effectiveness of blended learning in medical education.

Although prior studies have explored the general benefits and challenges of blended learning, limited research focuses on the interplay between self-regulatory learning, teacher credibility, and technological competence in medical education. Furthermore, the moderating role of perceived institutional support in enhancing these relationships remains underexplored. Therefore, this study aims to provide new insights into optimizing blended learning strategies for medical students by addressing these gaps. The following research questions guide the study.

(a) How does self-regulatory learning impact medical students’ academic performance in blended learning environments?
(b) How do perceived teacher credibility and technological competencies mediate the relationship between self-regulatory learning and student academic achievement?
(c) What is the moderating role of perceived institutional support in the relationships between self-regulatory learning, perceived teacher credibility, technological competencies, and academic performance?

This study contributes to understanding the impact of self-regulatory learning on medical students’ academic achievements in blended learning settings. It also advances knowledge by examining the mediating roles of teacher credibility and technological competence while exploring the moderating effect of perceived institutional support. By doing so, the research offers practical recommendations for enhancing blended learning outcomes in medical education.

## Theoretical framework and hypothesis development

### Behavioral learning theory

This paper is based on behavioral learning theory. Bandura (23) created behavioral learning theory to encourage intrinsic motivation and creative learning. In line with this, Anthonysamy et al. (24) initiated the student participation theory, describing that the physiological and psychological energy that participates in the learning process establishes the primary factor for students to learn. It suggests that factors such as students’ attitudes, emotions, and behaviors toward teachers affect the degree of student intention to use technology (25).

Behaviorist learning theory understands the “essence” of learning as learners showing specific behavioral responses to motivations (26). Learning occurs when the learner presents an expected or appropriate response to a certain momentum. This theory argues that teaching and learning refer to learners accepting instruction from teachers to achieve expected learning goals and externally manifesting expected learning behaviors. Therefore, learning based on biobehavioral learning theory belongs to receptive learning, and its learning basis is the metaphor of transmitting information from teachers to students. Teachers convey to students what students understand, allowing students to learn something.

To improve the influence of communication, teachers should design a learning environment to enhance the transmission of information. Teachers should construct a teaching environment that allows learners to respond to inducements as they think appropriate, thereby maximizing learners’ active actions, and learning is equivalent to changing the form or number of observed actions (5). Therefore, this study’s understanding of learning motivation is mainly based on biobehavioral learning theory (27), which provides students with learning stimulation through technology or strategies. Teachers can encourage or strengthen students’ desired learning behaviors in specific learning situations.

### Self-regulatory learning and student learning outcome

The growing use of Internet technologies in education has resulted in new learning paradigms, such as self-regulatory learning (28). Self-regulated learning is a multifaceted term that includes various aspects of human functioning, such as motivation, cognition, behavior, emotion, and metacognition (24). Many modern theorists consider an individual’s capacity to plan, implement, and consistently adapt or enhance various self-control strategies as a crucial aspect of the dynamic and flexible self-regulated learning process (29). It is challenging to propose a single definition for learning due to the various terminologies, such as e-learning, distributed learning, virtual learning, and distance learning over time. Müller et al. (4) have focused on creating an effective blended learning process that promotes positive outcomes.

Anthonysamy et al. (24) described self-regulatory learning through a blended learning approach comparing traditional face-to-face teaching and technology-mediated instructions. Hong et al. (30) referred to blended learning as a combination of classroom face-to-face studies with online learning. Similarly, Xu et al. (10) stated that blended learning is ‘the range of possibilities presented by combining Internet and digital media with established classroom forms that require the physical co-presence of teacher and students’. The digital media and resources blended into traditional classes may include audio or video streaming, wikis, online forums, web-based applications, collaboration and communication tools, and virtual learning environments (31). The use of self-regulatory learning is deemed to bring changes to teaching and learning patterns in higher education, including class flexibility, student commitment, control, and review of learning (7). Xu et al. (10) investigated blended education’s effect on students’ medical-related learning outcomes and indicated that blended learning has a positive impact on both student interest and academic achievement. Su et al. (7) found that students who participated actively in blended medical lessons and effectively used digital resources outperformed their less-engaged peers in terms of learning results. In their recent study, Wu et al. (3) examined the impact of blended learning on the skill development of medical students and suggested that students need to develop and improve their medical skills. Rasheed et al. (32) found that active participation in blended medical training programs led to enhanced technical abilities among students, indicating a beneficial effect of blended learning on skill acquisition. Li and Wang (33) emphasized the significance of teacher support and guidance in enhancing student learning outcomes in blended education. Yang et al. (34) highlight the importance of teachers in facilitating student engagement and performance in blended learning through timely feedback, instructional support, and the establishment of a conducive learning environment. This indicates that several factors, including technology access, available resources, teacher-student interaction, and motivation, impact the efficacy of medical education. We propose the following hypotheses for further investigation in this study.

*H1*: Self-regulated learning positively affects student learning outcomes under blended learning.

*H2*: Self-regulated learning is significantly related to technology competence under blended learning.

*H3*: Self-regulated learning is significantly related to teacher credibility under blended learning.

## Mediation mechanism

### Self-regulated learning, technological competencies, and student learning outcomes

Blended learning has become increasingly popular in academic settings due to its use of digital technologies and the Internet for educational purposes. Research has examined the correlation between self-regulated learning and student learning outcomes, resulting in a combination of positive and inconclusive results (33). A meta-analysis indicated that amalgamated learning yielded significant positive effects of moderate to large magnitude on student learning outcomes (35). The use of blended learning platforms may also improve students’ academic outcomes because of their adaptability and ease of use (36).

Additionally, technical capabilities impact the connection between self-regulatory learning and student learning results. Students who are well-versed in technology can better use digital learning opportunities, such as online discussion forums, group projects, and databases (6). Similarly, students’ technological competencies were positively related to their engagement and academic achievement in blended learning environments (32). The complex relationship between self-regulated learning, technological competence, and academic success must be acknowledged (13). Unequal access to technology and internet connection may lead to differences in students’ learning results, as Costello et al. (37) discovered. Instructional strategy, educator involvement, student motivation, and technological resources should be considered to provide everyone a quality and fair education. This study proposed the following hypothesis:

*H4*: Technological competence significantly mediates the relationship between self-regulated learning and student learning outcomes.

### Self-regulatory learning, perceived teacher credibility, and student learning outcome

Blended learning transforms the configuration of learning resources and realizes the transformation from an entity to a network environment (38). Self-regulatory learning through digital platforms helps students feel safe and secure (). How students see a teacher’s credibility might vary depending on factors, including their comfort level with blended learning and how they like to absorb information. Kuo and Tien (16) found that teachers’ trustworthiness strongly influenced student engagement and learning outcomes in blended courses. According to Müller et al. (4), instructors may boost their credibility and student satisfaction by improving their communication skills, topic knowledge, and response speed. A feeling of presence and social interaction fostered through digital interactions might enhance teachers’ credibility in the perception of their students. According to Xu et al. (10), instructors’ trustworthiness and students’ sense of care increase when instructors promptly and positively respond to students’ questions and concerns in blended learning environments. The following hypotheses were advanced in this investigation based on the prior literature:

*H5*: Teachers’ credibility mediates the relationship between self-regulated learning and student learning outcomes.

## Moderating mechanism

### Perceives institutional support as a moderator

Perceived institutional support refers to employees’ general belief that the organization respects their contributions and cares about their wellbeing (39). According to Bernarto et al. (40), institutional support protects individuals from the harmful effects of job stress. Perceived support serves an informational function by providing individuals with enough information to help define, comprehend, and cope with stressful events. It works as a social companionship function that satisfies the need to be accompanied and have affiliation and distracts individuals from stress (41). Perceived support serves an instrumental function that provides material resources and services needed to help cope with stress (11).

The support provided within an organization significantly influences the acquisition and advancement of technical skills (24). Jehanzeb (41) identified that perceived institutional support and collaborative learning positively correlated to students’ technological competencies within blended learning settings. This perspective supports empirical techniques like self-regulatory learning in top-down models, as opposed to qualitative interviews used in bottom-up models like the student approach to the learning model (10). Resource sharing, active participation in discussions, and assisting others can enhance technological competencies through exchanging knowledge and experiences (42). Wu et al. (3) found that well-designed blended learning platforms that promote social interactions and community building positively affect perceived institutional support and technological competencies. Versteijlen and Wals (15) identified a significant statistical correlation between perceived social support and technological competencies within blended learning environments. Additionally, Bamoallem and Altarteer (43) noted that students who receive support from organizations, engage in collaborative learning, and have access to well-designed blended learning environments are more likely to develop and demonstrate enhanced technological competencies.

**Figure 1:**
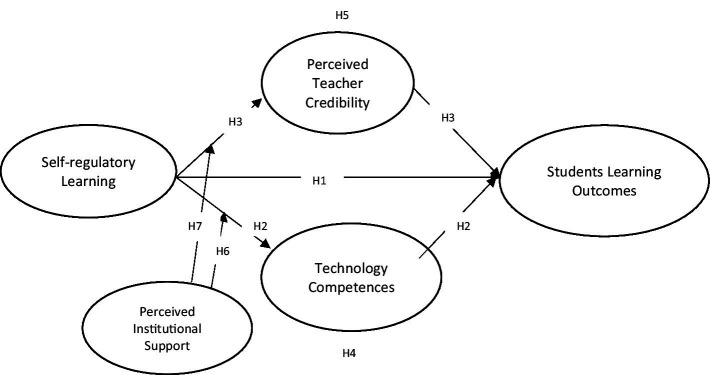
Study model.

In addition, there is a need to study the connection between teachers’ legitimacy and perceived institutional support. Perceived institutional support encompasses individuals’ belief in accessing supportive networks and resources, which can positively impact their wellbeing and academic achievements (44). Students’ evaluations of their instructors’ dependability, knowledge and qualifications are crucial in developing their credibility (10). Several studies have examined how students perceive their teachers’ credibility within blended learning environments and its relationship to their perceived institutional support. Finn et al. (45) found a significant positive connection between students’ evaluations of their teachers’ credibility and their perceptions of the teachers’ support. Additionally, Bruggeman et al. (46) highlighted a positive relationship between students’ evaluation of teachers’ institutional support and their credibility. In addition, this research suggests that the availability and quality of organizational learning resources positively influence students’ perceptions of teachers’ credibility in blended learning. Pishghadam et al. (47) identified that students’ evaluation of their teachers’ credibility, knowledge, competence, and communication abilities is higher when they perceive their teachers’ organizational support. In blended learning context, students who receive greater organizational support from their instructors are more likely to regard them as credible and trustworthy (48). The integration of online communication tools, along with thoughtful design considerations in blended learning environments, can substantially shape students’ perceptions of organizational support and their instructors’ credibility (see Figure 1). This study proposed the following hypotheses.

*H6*: Perceived institutional support moderates the relationship between self-regulatory learning and technological competencies.

*H7*: Perceived institutional support moderates the relationship between self-regulatory learning and perceived teacher credibility.

## Research methods

This study employed primary data from medical students in MBBS (Bachelor of Medicine, Bachelor of Surgery) programs at medical universities in Punjab province, Pakistan. The Punjab province, which is the most populous province in Pakistan, was selected purposely due to its medical universities and the researchers’ familiarity with the area. Moreover, purposive sampling was used to select MBBS students from six medical universities located in Punjab province. We used a cross-sectional questionnaire based on MBBS students’ self-administered survey. This study utilizes a convenience sampling method to reach the target participants with at least one month of blended learning experience. Additionally, convenience sampling, as a non-probability sampling method, allowed us to target participants considering their gender, education level, technology competence, and blended learning experiences (see Table 1). A total of 320 printed questionnaires were distributed to MBBS students in the last quarter of 2023, of which 275 questionnaires were returned with an 86% response rate.

**Table 1:** Participants information.

Students		
	Frequency	Percentage
Gender		
Male	150	54.5
Female	125	45.5
Age (in years)		
18–20	85	30.9
21–23	112	40.7
24–25	52	18.9
Above 25	26	09.5
Education (level)		
First-year	70	25.5
Second-year	80	29.1
Third year	65	23.6
Fourth year	60	21.8
Technological competencies		
Basic computer skills	120	43.6
Intermediate computer skills	115	41.8
Advanced computer skills	40	14.5
Blended learning experience		
1 to 3 months	40	14.5
3–6 months	130	47.3
6–9 months	75	27.3
More than 9 months	30	10.9

Before the formal survey, participants were asked for consent before participating in the study. All participants were informed about the purpose of the study, their participation was voluntary, their information would be kept confidential, and the collected data would be used for research purposes only. However, the participants were not rewarded for their participation in the study. Ethical approval for this study was obtained from the university review committee (Hunan University, code 202301). All relevant units of selected medical universities were approached, and permission was obtained for data collection.

### Measurement scale

The measurement scale comprises the instrument of study variables: blended learning, teachers’ credibility, perceived institutional support, technological competencies, and students’ learning outcomes. This study used adapted items from previous studies because they have been checked for reliability and validity (49). Seven items were adopted from the research instruments of Al-Omoush et al. (50) and Javier (51) to measure technological competencies. Ten items were adopted from Li-I Hsu (52) and Teven and McCroskey (53) to measure the participants’ perceptions of their teachers’ credibility. Eight items were adopted from San and Guo (54) to measure the perceived institutional support. Sixteen items were taken from Fuente et al. (55) to measure self-regulated learning, which includes three dimensions: planning (6 items), thoughtful learning (5 items), and study techniques (5 items). The learning outcomes were measured through 4 items from Ashraf et al. (18) using students’ self-reported academic performance (from the previous year). The adopted items were modified to be more relevant and applicable in blended teaching background. Meanwhile, based on data from students, as this study measured the student’s perceptions of their learning outcomes, the items were revised and adjusted accordingly. In addition, the study uses a 5-point Likert scale to measure the constructs, ranging from “strongly disagree” to “strongly agree.”

In addition, before the formal study, the designed questionnaire was shared with three experts to determine the suitability and validity of the constructs in the medical universities in Pakistan. The questionnaire was revised based on their comments and suggestions. Furthermore, a pilot test was conducted with 25 participants to ensure the legibility of the survey questionnaire. Small changes, such as in questionnaire terminology and language used, were made based on the feedback.

### Analysis

This study used the PLS-SEM (partial least rectangular structural equation modeling) method to analyze the data. PLS-SEM was chosen because it is appropriate for exploratory studies. The interpretation of PLS-SEM is also more straightforward and complex than that of covariance-based structural equation modeling (56, 57). SmartPLS was used to conduct the statistical analysis, and all the tests required for this research were performed with the help of this software. The direct effect, mediation, and moderation effects were tested to measure the relationships (58, 59).

## Results

### Participants background information

In total, 275 medical students enrolled in the medical universities participated in the study. Among the 275 participants, 150 (54.5%) were male and 125 (45.5%) were female students. Regarding the age range, 85 students (30.9%) were between 18–20 age, 112 (40.7%) were in 21–23 age, 52 (18.9%) were in 24–25 and 26 (09.5%) were above 25. In terms of the academic year, there were 70 first-year students (25.5%), 80 s-year students (29.1%), 65 third-year students (23.6%), and 60 fourth-year students (21.8%). Regarding technological competencies, 120 students (43.6%) reported having basic computer skills, 115 students (41.8%) indicated intermediate computer skills, and 40 students (14.5%) reported advanced computer skills. In terms of blended learning experience, 40 students (14.5%) had less than three months of experience, 130 students (47.3%) had three to six months of experience, 75 students (27.3%) had six to nine months of experience, and 30 students (10.9%) had more than nine months of online learning experience (Table 1).

### Measurement model

The evaluation of the measurement model included an assessment of discriminant validity, convergent validity, and internal consistency measures, as outlined by Kern et al. (60). According to Hair et al. (61), a minimum factor-loading criterion of 0.6 is recommended. The internal consistency of constructs was evaluated using measures such as Composite Reliability (CR) and Cronbach Alpha. All values of Cronbach Alpha and Composite Reliability (CR) significantly meet the minimum acceptable criterion of 0.70. Moreover, the Average Variance Extracted (AVE) values exceeded the minimum requirement of 0.5, indicating the convergent validity of constructs. Table 2 displays the significant findings, such as factor loading, CR, AVE, and Cronbach Alpha.

**Table 2:** Reliability and validity analysis.

Variables	Constructs	Factor loading	AVE	CR	α
Self-regulatory learning	SRL1	0.803	0.695	0.880	0.840
	SRL 2	0.720			
	SRL 3	0.758			
	SRL 4	0.776			
	SRL 5	0.786			
	SRL 6	0.788			
	SRL 7	0.714			
	SRL 8	0.713			
	SRL 9	0.827			
	SRL 10	0.723			
	SRL 11	0.702			
	SRL 12	0.876			
	SRL 13	0.784			
	SRL 14	0.756			
	SRL 15	0.826			
	SRL 16	0.886			
Perceived teacher credibility	PTC 1	0.835	0.648	0.859	0.793
	PTC 2	0.763			
	PTC 3	0.799			
	PTC 4	0.893			
	PTC 5	0.810			
	PTC 6	0.749			
	PTC 7	0.721			
	PTC 8	0.864			
	PTC 9	0.764			
	PTC 10	0.826			
Technologies competences	TC 1	0.752	0.738	0.788	0.721
	TC 2	0.713			
	TC 3	0.740			
	TC 4	0.706			
	TC 5	0.784			
	TC 6	0.758			
	TC 7	0.842			
Perceived institutional support	PIS 1	0.764	0.693	0.833	0.749
	PIS 2	0.702			
	PIS 3	0.846			
	PIS 4	0.742			
	PIS 5	0.826			
	PIS 6	0.804			
	PIS 7	0.786			
	PIS 8	0.790			
Students learning outputs	SLO 1	0.748	0.653	0.806	0.798
	SLO 2	0.832			
	SLO 3	0.790			
	SLO 4	0.768			

The discriminant validity of the model is determined through the Fornell–Larcker criterion and Heterotrait-monotrait (HTMT) (61). The ability of one variable to viably differentiate itself from another is known as its “discriminant validity.” Two different approaches, “Fornell-Larcker” and “cross-loading” statistical analysis, were used to determine the model’s “discriminant validity.” Table 3 illustrates that the square root of the AVE is higher than the correlation with other corresponding constructs (62). The top right diagonal indicates AVE’s square root, which is greater than all other corresponding construct correlations.

**Table 3:** Fornell & Larcker.

		1	2	3	4	5
1	Self-regulated learning	0.897				
2	Technological competency	0.383	0.861			
3	Teacher credibility	0.749	0.789	0.804		
4	Perceived institutional support	0.839	0.644	0.760	0.833	
5	Student learning outcome	0.730	0.743	0.662	0.689	0.805

Secondly, the discriminant validity is tested in this research using Heterotrait-monotrait (HTMT) ratio. The value HTMT ratios greater than 0.90 might be problematic for questioning discriminant validity (63). The findings in Table 4 show that all HTMT meet this limit (64), which confirms the discriminant validity of the research model.

**Table 4:** Heterotrait monotrait.

		1	2	3	4	5
1	Self-regulated learning	0.830				
2	Technological competency	0.621	0.807			
3	Teacher credibility	0.324	0.402	0.808		
4	Perceived institutional support	0.340	0.530	0.503	0.876	
5	Student learning outcome	0.201	0.630	0.230	0.503	0.850

### Structural model

The structural model was examined after measuring the constructs’ validity and reliability. As indicated in Table 5, the first step was to assess the constructs’ coefficient of determination (R^2^) and predictive relevance (Q^2^). Next, the hypotheses testing was carried out using standardized coefficients. Standardized path coefficients were utilized to test the hypotheses. Additionally, the overall quality of the model is improved by each structural path, as recommended by Henseler et al. (63). R^2^ values of 0.75, 0.50, and 0.25 for endogenous latent variables represent substantial, moderate, and weak effects, respectively,. According to the results, the R^2^ values for Teacher Credibility, Technological Competencies, and Learning Outcome are 0.628, 0.755, and 0.664, respectively, indicating a strong predictive power of the model (65, 66). Additionally, the Stone-Geisser test (Q2) was employed to assess the predictive value of the dependent variables. A Q^2^ value greater than zero is considered significant, while a Q^2^ value less than zero indicates the unreliability of the predictive value (67, 68). However, all of the constructs indicate that the model has strong predictive power, as suggested by (66, 69).

**Table 5:** Predictive accuracy.

Constructs	R^2^	Q^2^
Perceived teacher credibility	0.628	0.342
Technological competencies	0.755	0.540
Learning outcome	0.664	0.402

### Hypotheses testing

Following the assessment of goodness of fit, the hypotheses were further tested to determine the significance of the association. In this analysis, we use Bootstrapping at 5,000 with a replacement sample to assess the relative importance of associations (70). The study’s findings revealed that a significant relationship between self-regulated learning and student learning outcomes is supported by (*β* = 0.405, *t* = 2.784, *p* ≤ 0.05), showing a positive and significant relationship between self-regulated learning and student learning outcomes. The findings further revealed that self-regulated learning significantly impacts technological competence. The results show that self-regulated has a significant and positive impact on technological competence with (*β* = 0.856, *t* = 14.813, *p* ≤ 0.05), which approved H2. H3 shows that self-regulated learning significantly and positively influences teacher credibility, which is also supported by (*β* = 0.042, *t* = 2.085, *p* ≤ 0.05). Study findings support H1, H2, and H3, as shown in Table 6.

**Table 6:** Structural model evaluation.

	Hypotheses	*β*	*t*-value	*p*-value	Decision
H1	Self-regulated learning->Learning outcome	0.405	2.784	0.001	Supported
H2	Self-regulated learning->Technology competence	0.856	14.813	0.000	Supported
H3	Self-regulated learning->Teacher credibility	0.042	2.085	0.030	Supported

### Mediation analysis

The study employed teachers’ credibility and technological competencies to mediate the relationship between self-regulated learning and student academic performance. VAF technique was applied to measure mediation (71). Furthermore, the strength of this mediator was evaluated using the Variance Accounted for (VAF) method of estimating relative absorption suggested by Hair et al. (72). According to the VAF approach, VAF >80% show full mediation, 20%<VAF >80% show partial mediation, and less than 20% show no mediation. Nitzl et al. (73) added that particle mediation exists when there is a significant indirect and direct relationship between variables. The results illustrate that technological competencies partially mediate the relationship between self-regulated learning and student learning outcome as the direct effect (*β* = 0.405, *t* = 2.784, *p* ≤ 0.05) and indirect effect (*β* = 0.549, *t* = 6.455, *p* ≤ 0.05) with VAF 70.3% show partial mediation. We proposed the hypothesis that teachers’ credibility mediates the relationship between self-regulated learning and medical students’ performance. Again, the findings demonstrate that partial mediation of teachers’ credibility between self-regulated learning and medical student learning outcomes as the direct effect (*β* = 0.405, *t* = 2.784, *p* ≤ 0.05) and indirect effect (*β* = 0.220, *t* = 6.760, *p* ≤ 0.000) with VAF 68%. The results support H4 and H5 as shown in Table 7.

**Table 7:** Mediation analysis.

Relationship	Direct effect	Indirect effect	Total effect	VAF	Decision
Self-regulated learning->Technological competencies->Student learning outcome	*β* = 0.405, *T*-value = 2.784, *P*-value ≤0.05	*β* = 0.549, *T*-value = 6.455, *P*-value ≤0.05	*β* = 0.743, *p*-value = 0.000, *T*-value = 22.738	70.3%	Partial mediation
Self-regulated learning->Teachers credibility->Student learning outcome	*β* = 0.405, *T*-value = 2.784, *p*-value ≤0.05	*β* = 0.220, *p*-value ≤0.000, *T*-value = 6.760	*β* = 0.584, *p*-value = 0.000, *T*-value = 42.579	68%	Partial mediation

### Moderating analysis

The study further investigated the moderating influence of perceived institutional support to moderate the relationships between self-regulated learning, technological competencies, and teacher credibility. Table 8 shows that perceived institutional support moderates the relationship between self-regulated learning and teacher credibility with (*β* = 0.049 *t* = 3.128, *p* ≤ 0.05). The study findings confirm that perceived institutional moderate the relation between self-regulated and technological credibility with (*β* = 0.017, *t* = 2.765, *p* ≤ 0.05) support H8. Findings support H9.

**Table 8:** Moderation analysis.

Hypothesis testing	*β*	*T*-value	*p*-value	Decision
SRL × PIS->TC	0.049	3.128	0.041	Supported
SRL × PIS->TC	0.017	2.765	0.032	Supported

## Discussion

This study indicated that self-regulated learning significantly impacts student learning outcomes under the umbrella of blended learning. This finding relates to the idea that blended learning is comparable in effectiveness to traditional classroom-based learning (4). Su et al. (7) and Xu and Jaggars (74) also found that, on average, students who took online courses performed better academically than those who took conventional in-person courses. The research stressed the importance of course design and student peer support facilities in blended learning environments. This suggests the flexibility and convenience that online learning creates improve learning outcomes.

The study results further revealed that self-regulatory learning mediates the role of teachers’ credibility and technological competencies. Moreover, the study found that perceived institutional support moderates the relationship between self-regulation, perceived teacher credibility, and technological competencies. This was aligned with the fact students do better when they trust their instructors (13, 52).

The study analyzed adaptations in teachers’ levels of success concerning their credibility, competence, and kindness, and the findings indicated that teachers’ competence is significant for students’ academic achievement, followed by teachers’ likability and credibility. The study found that technological competence mediates the relationship between self-regulated learning and student learning outcomes, as Anthonysamy et al. (24) indicated. Numerous studies have also shown a strong correlation between students’ technological competence and learning outcomes in online courses (7). In line with this, the current study showed a favorable relationship between students’ technological competence, engagement in blended learning, and overall learning outcomes. In addition, the findings revealed that students’ technological competence was crucial in mediating the connection between their learning outcomes and blended courses. Students with good technological competence were able to engage more in blended classrooms and perform better in learning outcomes. This demonstrates that blended learning outcomes are positively correlated with students’ levels of technological competence.

Furthermore, the study results show that the association between self-regulated learning and perceived institutional support is moderated by the perceived degree of teacher credibility; i.e., when perceived institutional support is high, teacher’s credibility is high. Similarly, perceived institutional support positively correlated with student happiness and perceived learning outcomes. This highlights the value of perceived institutional support that might enhance learning when interacting with others online. Hill and Smith’s (11) study also indicates the correlation between blended course completion rates and students’ reports of perceived institutional support. Overall, students with perceived institutional support were expected to do well in blended courses and academic performance. This suggests that perceived institutional support significantly influences students’ participation and persistence in online education. Regarding the relationship between teachers’ credibility and student participation in blended learning, the study found that students’ perceptions of the teacher’s credibility are positively correlated with their course interest and satisfaction. This implies that students’ faith in their online teachers might significantly affect their learning outcomes.

## Conclusion

After the outbreak of COVID-19, there have been substantial changes in education, necessitating a swift shift to online instruction to maintain continuity in the face of the emergency. This trend was also found to be beneficial to higher education institutions as they can integrate both online and face-to-face teaching (17–19). Blended education is essential in improving students’ academic performance, notably medical students. However, this study indicates that blended learning is influenced by various factors, such as teaching credibility and technological competence. Moreover, a friendly learning climate is critical for fostering motivation, engagement, and overall academic accomplishment in the virtual setting. Teachers’ character, expertise, and resources might significantly affect students’ learning motivation and academic achievement in classrooms.

The study further argues that self-regulatory learning is vital forblended learning as it mediates the role of teachers’ credibility and technological competencies. This suggests that teachers’ credibility and technical capabilities are strongly associated with students’ learning experiences across subjects in medical education. Teachers’ technological competence also mediates the positive correlation between the blended learning environment and student academic performance and perceives teacher’s credibility. These factors become crucial in determining students’ active involvement and academic performance in blended courses. In addition, the study concluded that perceived institutional support is a significant mediator in the relationship between self-regulated learning and teachers’ credibility and technology competencies.

This study contributes to designing friendly and effective blended learning methodologies and support systems for students in medical sciences and other disciplines. It also helps teachers and other stakeholders create a friendly virtual classroom, therefore raising students’ academic performance. While the study’s primary population is medical students, its findings may have broader applicability and assist students of all majors and settings. It can help educational institutions to create a positive and encouraging blended learning environment by placing a premium on teacher credibility, technological proficiency, and the promotion of perceived institutional support.

### Limitation and future direction

This study provides insights into the factors that affect students’ academic performance in a blended learning environment. However, it is essential to acknowledge certain limitations. First, the study used convenient sampling methods in collecting the data, which might create a sampling bias. Furthermore, the study primarily examined undergraduate medical students with self-report measures, which may have been affected by some degree of response bias. Hence, future research should consider incorporating a more diverse sample to enhance the findings and broaden their generalizability. Moreover, including objective measures, such as academic performance data or observation of online interactions, could improve the evidence’s strength.

Future research could explore additional variables, such as student self-efficacy, parental involvement, and peer support, to further understand their impact on students’ academic achievement in blended learning. Longitudinal studies offer valuable insights into these variables’ enduring effects and dynamic interactions over an extended period. Additionally, it would be advantageous to investigate the efficacy of interventions or strategies to foster a positive blended learning environment and improve teacher proficiency.

## Data Availability

The raw data supporting the conclusions of this article will be made available by the authors, without undue reservation.
